# Advances and Challenges in Obstetric Intensive Care Medicine

**DOI:** 10.3390/jcm15041487

**Published:** 2026-02-13

**Authors:** Antonio Braga, Helder Konrad De Melo, Gabriela Paiva, Gustavo Mourão Rodrigues, Gustavo Yano Callado, Edward Araujo Júnior, Joffre Amim-Junior, Jorge de Rezende-Filho, Roberta Granese

**Affiliations:** 1 Department of Gynecology and Obstetrics, School of Medicine, Federal University of Rio de Janeiro (UFRJ), Rio de Janeiro 22240-003, RJ, Brazil; bragamed@yahoo.com.br (A.B.); gabrielapaivaslc@gmail.com (G.P.); joffre@me.ufrj.br (J.A.-J.); rezendef@me.ufrj.br (J.d.R.-F.); 2 Department of General and Specialized Surgery, School of Medicine and Surgery, Federal University of the State of Rio de Janeiro (UNIRIO), Rio de Janeiro 20271-062, RJ, Brazil; gustavo.rodrigues@unirio.br; 3 Postgraduate Program in Applied Health Sciences, University of Vassouras, Vassouras 27700-000, RJ, Brazil; 4 Intensive Care Unit, Gloria D’or Hospital, Rede D’or, Rio de Janeiro 22211-230, RJ, Brazil; helder.melo@gmail.com; 5 Discipline of Woman Health, Albert Einstein Israelite College of Health Sciences, Albert Einstein Israelite Hospital, São Paulo 05652-900, SP, Brazil; gycallado@gmail.com; 6 Discipline of Woman Health, Municipal University of São Caetano do Sul (USCS), São Caetano do Sul 09521-160, SP, Brazil; araujojred@terra.com.br; 7 Department of Obstetrics, Paulista School of Medicine, Federal University of São Paulo (EPM-UNIFESP), São Paulo 04023-062, SP, Brazil; 8 Department of Biomedical and Dental Sciences and Morphofunctional Imaging, “G. Martino” University Hospital, 98100 Messina, Italy

**Keywords:** obstetric critical care, maternal–fetal physiology, severe maternal morbidity, intensive care unit, multidisciplinary care

## Abstract

Obstetric critical care encompasses the management of pregnant and postpartum women with life-threatening conditions, requiring integration of intensive care principles with pregnancy-specific physiological, ethical, and organizational considerations. Although pregnancy is a physiological state, profound maternal adaptations may mask early signs of clinical deterioration, allowing rapid progression to a critical illness condition. This review provides a comprehensive overview of the foundations of obstetric intensive care, addressing maternal–fetal physiology, recognition of severity, organ support strategies, and contemporary models of care. Key aspects discussed include cardiovascular, respiratory, renal, and hematological adaptations of pregnancy; principles of airway management and mechanical ventilation; hemodynamic support; transfusion strategies guided by viscoelastic testing; renal replacement therapy; extracorporeal support, including extracorporeal membrane oxygenation and cardiopulmonary bypass; and the safe use of diagnostic imaging involving ionizing radiation. The role of point-of-care ultrasonography, structured early warning systems, and advanced monitoring in early detection and management of clinical deterioration is emphasized. Special attention is given to maternal–fetal interactions, fetal monitoring in the intensive care unit (ICU), and complex decision-making regarding timing and mode of delivery. The review also highlights the importance of multidisciplinary and multiprofessional collaboration, ethical challenges inherent to dual-patient care, and emerging strategies to expand access to specialized care, including tele–ICU models and artificial intelligence–assisted surveillance. Across all scenarios, maternal stabilization remains the primary determinant of fetal outcome. A structured approach grounded in maternal–fetal physiology and ethical principles is essential to reduce preventable maternal and perinatal morbidity and mortality in high-complexity settings.

## 1. Introduction

Obstetric critical care is a highly complex field that integrates obstetrics, internal medicine, anesthesiology, and intensive care [1]. Although pregnancy is physiological, it induces systemic adaptations that alter disease presentation, laboratory findings, and treatment responses, often reducing maternal reserve and masking early signs of severity, thereby facilitating rapid progression to critical illness.

In recent decades, the obstetric population has become increasingly vulnerable due to advanced maternal age, a higher prevalence of chronic diseases, and greater use of assisted reproductive technologies. Simultaneously, advances in critical care have improved survival in severe maternal conditions. Consequently, intensive care unit (ICU) admission during pregnancy or the puerperium represents both a marker of severity and an opportunity for timely intervention to prevent severe maternal morbidity and mortality, which remain largely preventable [2].

The main indications for obstetric ICU admission include severe hemorrhage, hypertensive disorders, sepsis, acute respiratory failure, thromboembolism, anesthetic complications, and decompensation of preexisting conditions [3]. These conditions often progress rapidly, and diagnosis may be delayed because early symptoms overlap with normal pregnancy changes [4].

Management of critically ill obstetric patients requires adaptation of standard intensive care principles to pregnancy-specific physiology and fetal considerations. Despite limited obstetric-specific evidence, maternal stabilization remains the primary goal, as fetal outcomes depend directly on maternal status [2]. In addition to clinical challenges, care involves complex ethical, emotional, and organizational decisions, highlighting the need for early recognition, multidisciplinary coordination, and specialized training. This article reviews key aspects of obstetric intensive care to support improved maternal and perinatal outcomes.

## 2. Epidemiology of Severe Maternal Morbidity

Understanding the epidemiology of critical illness in pregnancy and the puerperium is essential for healthcare planning and prevention [5,6]. Traditional focus on maternal mortality underestimates disease burden, as most severe complications do not result in death [3]. Instead, maternal mortality represents only the “tip of the iceberg,” while many women survive life-threatening conditions after ICU admission, constituting maternal near miss [7].

Obstetric critical illness spans a continuum from early deterioration to organ failure and is pragmatically defined by significant organ dysfunction requiring intensive monitoring or life support due to high risk of severe morbidity or death [3]. ICU admission rates range from 0.07% to 0.89% of pregnancies, with higher rates in tertiary centers reflecting referral of high-risk cases [8]. Although uncommon, obstetric ICU admission is associated with substantial severity, with maternal mortality around 8%, particularly higher in low- and middle-income countries due to inequities in access and delays in care. Hypertensive disorders and obstetric hemorrhage account for more than half of ICU admissions, followed by sepsis, severe respiratory complications, cardiac disease, thromboembolism, and decompensation of preexisting conditions.

Risk factors include advanced maternal age, limited prenatal care, chronic comorbidities, and racial and socioeconomic inequities; Black women experience disproportionately higher rates of severe morbidity and mortality, reflecting structural determinants of health [9,10]. Critical maternal illness is also associated with adverse perinatal outcomes, including fetal and neonatal death, underscoring the need for integrated multidisciplinary care. In summary, although rare, critical illness in pregnancy has major maternal and perinatal implications. ICU-based epidemiological data are essential to identify risk patterns, care gaps, and opportunities for early intervention to reduce preventable morbidity and mortality.

## 3. Pregnancy-Induced Physiological Changes

Pregnancy induces widespread physiological adaptations that support fetal growth but substantially alter disease presentation, laboratory interpretation, and therapeutic responses [11]. In obstetric critical care, failure to recognize these changes may lead to diagnostic delay and inappropriate management. Cardiovascular adaptations include a 30–50% increase in cardiac output with reduced systemic vascular resistance and a mid-gestation decline in mean arterial pressure. These changes may mask early hypovolemia or shock, allowing significant blood loss before hypotension becomes apparent [12]. Compression of the inferior vena cava by the gravid uterus can further reduce venous return, particularly in the supine position, necessitating lateral uterine displacement during resuscitation and invasive procedures [12].

Respiratory adaptations include reduced functional residual capacity, increased oxygen consumption, and progesterone-mediated hyperventilation, resulting in compensated respiratory alkalosis. Consequently, pregnant patients have limited respiratory reserve and are prone to rapid hypoxemia during critical illness [13]. Hematological changes include physiological anemia and a hypercoagulable state, increasing thromboembolic risk, especially in the presence of immobility or sepsis. Renal plasma flow and glomerular filtration rate increase, lowering baseline creatinine levels; values considered normal outside pregnancy may therefore indicate renal dysfunction. Gastrointestinal and hepatic changes increase aspiration risk and complicate the interpretation of liver enzyme abnormalities, while endocrine and metabolic adaptations heighten vulnerability to stress-related metabolic disturbances.

Overall, pregnancy creates a unique physiological context in which traditional severity markers may be misleading, as shown in Table 1. Recognition of these adaptations is essential to distinguish physiology from pathology and to guide appropriate monitoring and management of critically ill pregnant patients.

## 4. Pregnancy-Related Pharmacokinetic and Pharmacodynamic Changes and Implications for Intensive Care Management

Pregnancy induces deep physiologic adaptations that significantly alter both pharmacokinetics (PK) and pharmacodynamics (PD), with important implications for drug dosing and therapeutic monitoring in the intensive care unit. These changes are dynamic across gestation and extend into the immediate postpartum period, when rapid physiologic reversal may further complicate drug exposure [1].

Maternal plasma volume increases by approximately 40–50%, accompanied by expansion of total body water and increased adipose tissue. These changes result in an increased volume of distribution for many hydrophilic drugs (e.g., β-lactam antibiotics) and may also affect lipophilic agents (e.g., sedatives and anesthetics). Consequently, standard loading doses may be insufficient to achieve therapeutic plasma concentrations, particularly in critically ill pregnant patients with sepsis or shock, in whom capillary leak and third spacing further expand the effective distribution volume [2].

Glomerular filtration rate and renal plasma flow increase early in pregnancy and remain elevated until late gestation. Drugs predominantly eliminated by renal excretion may therefore exhibit enhanced clearance and reduced exposure, particularly when administered by intermittent dosing. This is clinically relevant for several antibiotics and other renally cleared agents, supporting the use of optimized dosing strategies (e.g., higher doses, shortened intervals, or prolonged/continuous infusions) guided by clinical response and, when available, therapeutic drug monitoring [11].

Pregnancy is associated with selective modulation of hepatic enzyme activity. Cytochrome P450 isoenzymes such as CYP3A4 and CYP2D6 generally demonstrate increased activity, leading to accelerated metabolism of their substrates, whereas other pathways may be unchanged or reduced. These alterations can lower plasma concentrations of certain sedatives, analgesics, and cardiovascular drugs, increasing the risk of subtherapeutic exposure if standard non-pregnant dosing is applied. Interindividual variability is substantial, reinforcing the need for careful titration to clinical effect [11].

For antimicrobial therapy, pregnancy-related PK changes may compromise target attainment, particularly for time-dependent antibiotics. In critically ill obstetric patients, underexposure may contribute to treatment failure. Dosing strategies should prioritize achieving adequate maternal serum concentrations, as maternal stabilization and infection control are the primary determinants of fetal well-being. When feasible, extended or continuous infusions and therapeutic drug monitoring should be considered [11].

Altered volume of distribution and hepatic metabolism may affect both onset and duration of sedative and analgesic agents. Clinicians should anticipate variable responses, avoid fixed dosing schemes, and titrate medications to the lowest effective dose while closely monitoring maternal hemodynamics, level of consciousness, and ventilation [12].

Physiologic reductions in systemic vascular resistance during pregnancy may modify vasopressor responsiveness. Importantly, vasopressors should not be withheld when indicated for maternal hypotension or shock. Adequate maternal perfusion pressure is essential to preserve uteroplacental blood flow, and fetal considerations should not delay timely hemodynamic support. Drug selection and titration should be guided by maternal hemodynamic targets, recognizing that restoring maternal stability remains the most effective strategy for fetal protection [12].

In the immediate postpartum period, rapid shifts in volume status and organ function may lead to abrupt changes in drug disposition. Close reassessment of dosing is therefore essential to avoid toxicity or loss of efficacy during this transition.

## 5. Maternal–Fetal Blood Gas Physiology

Understanding maternal–fetal blood gas physiology is central to the management of critically ill pregnant patients, as fetal oxygenation and carbon dioxide elimination depend directly on maternal ventilation, hemodynamics, and uteroplacental circulation. Even minor alterations in maternal blood gas parameters may result in disproportionate fetal acid–base disturbances, particularly in critical illness [12,13].

During normal pregnancy, increased tidal volume and minute ventilation lead to a mild physiological respiratory alkalosis, with maternal PaCO_2_ values around 28–32 mmHg. This reduction is essential to maintain the gradient required for fetal CO_2_ elimination. Maternal PaO_2_ generally remains normal or slightly increased. Oxygen transfer occurs exclusively by placental diffusion and depends on maternal PaO_2_, uteroplacental blood flow, and the properties of fetal hemoglobin, which has a higher affinity for oxygen. The double Bohr effect further facilitates oxygen transfer by promoting maternal oxygen release and fetal oxygen uptake [14].

Fetal blood gases differ physiologically from maternal values, with a low PaO_2_ (approximately 20–30 mmHg) but adequate oxygen saturation due to fetal hemoglobin, and a slightly lower pH reflecting active metabolism. In maternal critical illness—such as respiratory failure, shock, or sepsis—hypoxemia and hypercapnia rapidly impair placental gas exchange, leading to fetal hypoxia and acidosis. In mechanically ventilated pregnant patients, gas exchange targets should respect gestational physiology. Permissive hypercapnia should be used cautiously, and maternal PaCO_2_ is generally recommended to be maintained below 45–50 mmHg. Maternal stabilization remains the most effective strategy for fetal protection.

## 6. Fluid and Electrolyte Balance in the Critically Ill Obstetric Patient

Pregnancy is characterized by plasma volume expansion (40–50%), physiological sodium and water retention, and reduced systemic vascular resistance. While these adaptations ensure adequate uteroplacental perfusion, they increase vulnerability to volume disturbances during critical illness [15]. Assessment of volume status is challenging, as isolated clinical signs are often unreliable. Dynamic methods, including echocardiography and fluid responsiveness assessment, are preferred. Both hypovolemia, which compromises placental perfusion, and fluid overload, which increases the risk of pulmonary edema—particularly in preeclampsia and sepsis—should be avoided [1].

Isotonic crystalloids are first-line fluids for resuscitation and maintenance, whereas colloids should be used cautiously. Electrolyte disturbances are frequent, including hyponatremia (excess free water, oxytocin), potassium abnormalities (vomiting, acidosis, renal dysfunction), and calcium and magnesium disturbances, particularly related to massive transfusion and magnesium sulfate therapy [16]. Correction strategies should prioritize maternal stabilization, which remains the most effective means of preserving fetal well-being [17,18].

## 7. Recognition of Severity in the Obstetric Population

Early recognition of the critically ill pregnant or postpartum patient is a key determinant of reduced maternal morbidity and mortality. Physiological adaptations of pregnancy may mask early signs of deterioration, allowing rapid and silent progression of disease [19,20,21].

Recognition of severity in the obstetric population requires awareness that many of the physiological adaptations of pregnancy may blur the distinction between normal gestational changes and early critical illness. Common diagnostic pitfalls, such as attributing tachycardia, leukocytosis, dyspnea, or preserved blood pressure to “physiologic pregnancy”, may delay recognition of deterioration and contribute to preventable morbidity. Table 2 summarizes these frequent misinterpretations and their practical implications for intensive care decision-making, highlighting how subtle abnormalities and abnormal trajectories, rather than isolated values, should prompt early investigation and escalation of care in pregnant and postpartum patients [1,2,11,12].

Significant blood loss may occur before hypotension becomes apparent. Persistent tachycardia, altered mental status, oliguria, and peripheral hypoperfusion should raise concern even in the presence of apparently normal blood pressure [12]. Reduced respiratory reserve makes pregnant patients particularly susceptible to hypoxemia; tachypnea and increasing oxygen requirements are early warning signs. Neurological, laboratory, and subtle signs of sepsis require prompt investigation. In pregnancy, minor laboratory deviations may reflect clinically significant organ dysfunction. Delayed recognition of obstetric sepsis is consistently associated with worse outcomes [22].

Early warning systems such as the Modified Early Obstetric Warning System (MEOWS) support the detection of clinical deterioration using gestation-adjusted parameters. Two or more moderate alert criteria warrant immediate evaluation, while any high-risk criterion requires urgent response and potential escalation of care. MEOWS is an adjunct and does not replace clinical judgment (Table 3). Continuous vigilance, frequent reassessment, and effective multidisciplinary communication are essential. Early identification of critical illness reduces progression to multiple organ failure and is a major determinant of maternal and fetal survival in high-complexity settings [11,23].

## 8. Severe Obstetric Versus Non-Obstetric Conditions

The assessment of critically ill obstetric patients requires careful distinction between conditions directly related to pregnancy and those of non-obstetric origin, as this differentiation guides diagnostic reasoning, therapeutic strategies, and decisions regarding the timing and mode of delivery. Overlapping clinical manifestations between these entities represent a frequent challenge in obstetric critical care (Table 4).

Obstetric conditions include hypertensive disorders of pregnancy, obstetric hemorrhage, obstetric sepsis, amniotic fluid embolism, acute fatty liver of pregnancy, and HELLP syndrome. In these settings, resolution of pregnancy may modify disease progression and often forms part of the therapeutic strategy; however, delivery should never precede maternal stabilization [26].

In contrast, non-obstetric conditions comprise medical or surgical diseases independent of pregnancy, such as non-obstetric sepsis, pneumonia, severe asthma, pulmonary embolism, cardiac disease, acute kidney injury, pancreatitis, trauma, and neurological disorders. Although influenced by gestational physiology, their course is rarely altered by delivery, which should be considered only when there is a clear obstetric indication or when maternal status precludes safe continuation of pregnancy [27].

Distinguishing between obstetric and non-obstetric conditions is not always straightforward, as signs such as hypertension, abdominal pain, dyspnea, laboratory abnormalities, and neurological symptoms may occur in both contexts. Therefore, initial management should prioritize maternal stabilization and organ support, with parallel diagnostic investigation and frequent reassessment. Pregnancy termination should be considered a therapeutic intervention only when maternal or fetal benefit is evident or when clinical deterioration persists despite adequate support [28].

## 9. Congenital Heart Disease in Pregnancy: Intensive Care Considerations

Advances in pediatric cardiology and cardiac surgery have resulted in a growing population of women with congenital heart disease (CHD) reaching reproductive age, making CHD an increasingly important contributor to maternal morbidity and ICU admission. Pregnancy imposes substantial cardiovascular stress, including increased plasma volume, cardiac output, and heart rate, which may exceed the compensatory capacity of certain congenital lesions and predispose to decompensation [3,4].

Risk stratification is central to the management of pregnant patients with CHD and should ideally occur preconception or early in pregnancy. Lesion-specific anatomy, baseline functional status, ventricular function, pulmonary vascular resistance, prior surgical repair, and history of arrhythmia or heart failure all influence maternal risk. Patients with complex physiology—such as single-ventricle circulation, Eisenmenger syndrome, severe pulmonary hypertension, systemic right ventricle, or significant ventricular dysfunction—are at particularly high risk for critical illness [7].

Common triggers for decompensation include the hemodynamic burden of late gestation, acute volume shifts, anemia, infection, hypertensive disorders, arrhythmias, thromboembolic events, and the rapid physiologic changes associated with delivery and the immediate postpartum period. Notably, clinical deterioration frequently occurs postpartum, when autotransfusion from uterine involution and mobilization of extravascular fluid abruptly increase preload and may overwhelm limited cardiac reserve [20].

ICU management priorities in pregnant or postpartum patients with CHD include meticulous attention to oxygenation, preload and afterload balance, and rhythm control. Both hypovolemia and fluid overload may precipitate instability, necessitating cautious, individualized fluid strategies guided by invasive or noninvasive hemodynamic assessment. Arrhythmia surveillance is essential, as atrial and ventricular arrhythmias are common precipitants of acute decompensation. Anticoagulation poses additional challenges, requiring careful balancing of maternal thrombotic risk, bleeding risk, and timing of delivery [7,20].

Optimal care requires close multidisciplinary coordination among critical care, cardiology (ideally adult congenital heart disease specialists), maternal–fetal medicine, anesthesia, and neonatology. Decisions regarding timing and mode of delivery, invasive monitoring, and escalation of support should be individualized and revisited dynamically as maternal physiology evolves. Early ICU involvement is recommended when signs of cardiovascular instability emerge, rather than delaying escalation until overt failure occurs.

As survival continues to improve for women with congenital heart disease, preparedness for their management in obstetric critical care settings is increasingly essential. Recognition of high-risk physiology, anticipation of decompensation, particularly in the postpartum period, and coordinated multidisciplinary care are key determinants of maternal and perinatal outcomes.

## 10. Severity Scores and Indications for Intensive Care Unit Admission

Early transfer of pregnant or postpartum women to the ICU, before the onset of multiple organ failure, is associated with improved maternal and perinatal outcomes, supporting a deliberately low threshold for escalation of care [29]. While clinical judgment remains paramount, objective scoring systems help standardize decision-making and facilitate communication among teams.

The Modified Early Obstetric Warning System (MEOWS) is an obstetric-specific tool designed for early detection of clinical deterioration, serving as a trigger for prompt reassessment and escalation of care. The presence of two or more moderate alert parameters or any severe alert criterion warrants immediate evaluation and consideration of ICU admission [30].

The Sequential Organ Failure Assessment (SOFA), although not obstetric-specific, plays a key role in assessing established organ dysfunction, particularly in sepsis, respiratory failure, and hemodynamic instability. A SOFA score ≥ 2, especially when associated with clinical deterioration or increasing organ support requirements, should prompt ICU admission. Serial increases in SOFA reflect worsening organ dysfunction and mandate urgent escalation of care (Table 5).

Early recognition and timely management of sepsis in obstetric patients remain major determinants of maternal and perinatal outcomes. However, the direct application of non-pregnant sepsis criteria, including Sepsis-3 definitions, carries important limitations in pregnancy. Physiologic adaptations such as baseline tachycardia, leukocytosis, increased cardiac output, and altered blood pressure thresholds may lead to misclassification, either by overestimating severity or by masking true deterioration due to the high compensatory reserve of pregnancy. These limitations underscore the need for obstetric-adjusted approaches to early sepsis identification [21,22].

Obstetric-specific early warning tools, such as the Sepsis in Obstetrics Score (SOS), have been proposed to improve early suspicion and triage of septic pregnant and postpartum patients [30,32]. The SOS incorporates maternal vital signs and laboratory parameters interpreted within the context of pregnancy-related physiological changes, facilitating earlier recognition of abnormal trajectories rather than reliance on static thresholds. While not intended to replace clinical judgment, such tools may support timely escalation of care, prompt initiation of antimicrobials, and appropriate ICU referral.

Beyond early recognition, management of obstetric sepsis must be driven by the underlying etiology, with particular emphasis on timely source control. In cases of suspected intrauterine infection or chorioamnionitis, prompt antimicrobial therapy alone may be insufficient without decisive source control. When maternal condition deteriorates or fails to improve, expedited delivery, after multidisciplinary discussion involving obstetrics, critical care, anesthesia, and neonatology, often represents a critical component of treatment. The timing and mode of delivery should be individualized, balancing maternal stabilization, gestational age, and fetal status [30,32].

Other common sources of obstetric sepsis include urinary tract infection, pneumonia, surgical site infection, and complications related to retained products of conception or postpartum uterine infection. For each, early identification of the infectious focus and rapid implementation of targeted source control measures are essential and should occur in parallel with early broad-spectrum antimicrobial therapy, hemodynamic resuscitation, and organ support [21].

In obstetric sepsis, maternal stabilization remains the primary therapeutic priority, as improvements in maternal perfusion, oxygenation, and metabolic status are the most effective means of preserving fetal well-being. Integrating obstetric-specific early recognition tools with etiology-focused management and multidisciplinary coordination is therefore essential to reduce delays in diagnosis and improve outcomes in this high-risk population.

No scoring system replaces clinical judgment. Active hemorrhage, suspected sepsis, impending respiratory failure, acute neurological changes, or concern expressed by the care team justify ICU admission regardless of score values [32].

## 11. Unanticipated Intensive Care Unit Admission in the Immediate Postpartum Period

The immediate postpartum period represents the highest-risk window for unanticipated ICU admission in obstetric patients, despite the frequent perception that delivery marks the resolution of pregnancy-related risk. Rapid and profound physiological changes occur after childbirth, including autotransfusion from uterine contraction, mobilization of extravascular fluid, abrupt shifts in preload and afterload, and dynamic neurohormonal adjustments. These transitions may precipitate acute decompensation even after an apparently uncomplicated pregnancy and delivery [1].

The most common causes of unexpected postpartum ICU admission include postpartum hemorrhage, hypertensive complications (including severe preeclampsia and eclampsia), sepsis, venous thromboembolism, peripartum cardiomyopathy, and complications related to anesthesia or operative delivery. Importantly, multiple processes may coexist, and early manifestations are often subtle. Tachycardia, mild hypotension, dyspnea, altered mental status, or laboratory abnormalities may precede rapid deterioration, underscoring the importance of heightened surveillance during this vulnerable period [4].

Recognition of “red flag” trajectories is critical. Failure to stabilize after initial hemorrhage control, escalating oxygen requirements, refractory hypertension, worsening renal or hepatic dysfunction, or unexplained respiratory or neurologic symptoms should prompt early escalation of care. The postpartum patient’s preserved physiologic reserve may mask severity until abrupt collapse occurs, contributing to delayed ICU referral if reliance is placed solely on static thresholds [4].

Effective management of postpartum critical illness requires anticipatory monitoring, early multidisciplinary involvement, and a low threshold for ICU admission when instability is suspected. Structured early warning systems adapted to the postpartum context, coupled with timely source control, hemodynamic support, and organ-specific management, are essential to reduce morbidity and mortality. Recognizing the postpartum period as a distinct phase of risk, rather than a continuation of pregnancy, has important implications for both clinical vigilance and systems of care [1].

## 12. Inter- and Intra-Hospital Transfer in Obstetric Critical Care

Transfer of critically ill pregnant patients, whether between hospitals or within the same institution, is a complex therapeutic intervention requiring structured planning, effective communication, and absolute prioritization of maternal stability. Ideally, critically ill obstetric patients should be managed in centers offering obstetric services, adult ICU care, advanced neonatal support, and blood bank availability [33].

Prior to transfer, maternal stabilization is mandatory, with clear documentation of hemodynamic, respiratory, and neurological status. Fetal assessment, when feasible, should not delay transport [34,35]. During transfer, continuous monitoring, adequate venous access, secure fixation of devices, and left uterine displacement should be ensured. When there is a high likelihood of respiratory deterioration, endotracheal intubation should be performed before transport [36].

Fetal monitoring during transfer may be considered selectively but should not delay transport, as fetal deterioration usually reflects maternal instability. Intra-hospital transfers also represent high-risk periods and require direct communication among obstetric, intensive care, anesthesia, and nursing teams [37].

## 13. Adult ICU Versus Obstetric ICU: Models of Care

Multiple care models coexist, including admission to general adult ICUs, specialized obstetric ICUs, and hybrid shared-care models. The choice of setting should consider maternal disease severity, need for advanced organ support, fetal viability, institutional resources, and team expertise [38,39].

Adult ICUs are best equipped to manage multiple organ failure, advanced mechanical ventilation, invasive hemodynamic support, and renal replacement therapy, and are often the safest environment for patients with shock, severe sepsis, acute respiratory distress syndrome, or neurological dysfunction. However, limitations include reduced familiarity with gestational physiology and fetal surveillance, necessitating close integration with obstetric teams [40].

Obstetric ICUs or high-dependency obstetric units are suitable for patients requiring intensive monitoring without advanced multiorgan support. Their strengths include expertise in pregnancy-specific physiology and complications and closer integration with perinatal care. Their main limitation is reduced capacity to manage rapid clinical deterioration, requiring timely transfer to an adult ICU when necessary [25,40].

Hybrid models combine obstetric expertise with the technical resources of adult ICUs and may reduce communication failures, but depend on clear protocols and collaborative institutional culture. Regardless of the model, maternal stabilization must remain the priority, as fetal outcome is intrinsically linked to maternal condition. Excellence in care relies not on the physical location but on multidisciplinary integration, readiness to escalate care, and institutional capacity to provide advanced maternal support while addressing obstetric and fetal needs [40,41,42].

## 14. Core Principles of Managing the Critically Ill Obstetric Patient

Initial assessment of critically ill pregnant or postpartum patients should follow the same structured approach used in non-pregnant critically ill adults, prioritizing airway, breathing, and circulation (ABC), immediate stabilization, and rapid referral to the appropriate level of care. Care should be delivered within a defined critical-care pathway, with clear team responsibilities and escalation flows.

Obstetric critical care can be conceptualized in graded levels. Level 0 corresponds to ward-based care. Level 1 includes patients at risk of deterioration or recently stepped down who require increased surveillance. Level 2 includes patients requiring invasive monitoring or specialized interventions with support for a single organ system, excluding advanced ventilatory support. Level 3 includes patients requiring invasive mechanical ventilation or respiratory support plus at least one additional organ-support modality. Level 4 involves highly specialized therapies such as extracorporeal membrane oxygenation (ECMO) or advanced circulatory assist devices [43].

Care delivery should integrate three core components: early recognition, coordinated response, and provision of advanced-level support. Level 2 care may occur in advanced obstetric areas or intermediate units, including mobile maternal critical care concepts, whereas Levels 3–4 should occur in an ICU. Organ dysfunction varies by etiology; lung involvement is most common, followed by hematologic, cardiovascular, renal, and neurologic dysfunction, often progressing to multiorgan failure. Maternal stabilization remains the primary determinant of fetal well-being; in general, interventions that benefit the mother benefit the fetus [44].

Airway management is a priority, as pregnant patients desaturate rapidly and have an increased risk of a difficult airway. Units caring for critically ill obstetric patients should have advanced airway devices (e.g., second-generation supraglottic airways, videolaryngoscopy, and cricothyrotomy equipment) and trained teams. Respiratory support should ensure adequate gas exchange; high-flow systems and non-invasive ventilation may be used selectively, but failure or severity criteria should prompt timely intubation and lung-protective ventilation. Targets should maintain adequate oxygenation and relative normocapnia, as permissive hypercapnia is less tolerated due to impaired placental CO_2_ clearance. In refractory hypoxemia (e.g., severe acute respiratory distress syndrome—ARDS), advanced strategies—including prone positioning, selective delivery, and ECMO—may be considered with multidisciplinary and family counseling [43].

Circulatory management aims to preserve uteroplacental perfusion. Hemorrhagic, septic, and cardiogenic shock should be treated early and aggressively, with large-bore vascular access, invasive monitoring when indicated, and ultrasound guidance when feasible. After 20 weeks, left uterine displacement should be maintained to optimize venous return. Fluid resuscitation, blood products, and vasoactive agents should follow established protocols without undue hesitation, as maternal instability poses greater fetal risk. Nutritional support should not be overlooked; pregnancy is relatively hypercatabolic and ketosis may develop early during fasting, favoring enteral nutrition whenever possible. ECMO (veno-venous or veno-arterial) has been successfully used in pregnancy and the puerperium and requires specialized teams and careful multidisciplinary planning, including delivery timing and mode when relevant [43]. Support of other organ systems follows general critical care principles with pregnancy-specific interpretation, coupled with rigorous infection prevention and continuous audit to improve care.

## 15. Common Challenges of Peripartum Intubation

Peripartum tracheal intubation is high-risk in obstetric anesthesia and critical care. Pregnancy-related anatomic and physiological changes, together with frequent comorbid pathology, increase the incidence of difficult airway, rapid hypoxemia, and induction-related complications. Anticipation and pregnancy-adapted strategies are essential to reduce maternal and fetal morbidity [45].

Aspiration risk is increased due to delayed gastric emptying, increased intra-abdominal pressure, and reduced lower esophageal sphincter tone; therefore, all pregnant patients should be treated as having a “full stomach.” Prophylaxis may include intravenous H_2_ blockers or proton pump inhibitors when feasible, sodium citrate immediately before induction, and gastric aspiration if a nasogastric tube is in place. Head-up positioning, appropriate cricoid pressure, and avoiding excessive mask ventilation pressures help reduce regurgitation and aspiration [46].

Rapid desaturation is expected because functional residual capacity is reduced and oxygen consumption increased. Preoxygenation should be optimized; high-flow nasal oxygen may be considered for preoxygenation and apneic oxygenation, with caution in infectious-risk settings due to aerosol generation. In respiratory failure, rapid-sequence induction may require modification, allowing cautious manual ventilation under cricoid pressure to prevent severe hypoxemia [47].

Difficult intubation is more frequent due to upper-airway edema, weight gain, breast enlargement, and reduced cervical mobility. Airway management should follow obstetric-specific guidance (e.g., Obstetric Anaesthetists’ Association/Difficult Airway Society), with a structured failed-intubation/failed-oxygenation plan and immediate access to videolaryngoscopy, second-generation supraglottic devices, and emergency surgical airway equipment. “Ramped” positioning improves laryngoscopic view, particularly in patients with higher body mass index.

In hypertensive disorders, especially severe preeclampsia, the pressor response to laryngoscopy increases the risk of intracranial hemorrhage; strict blood pressure control before induction is mandatory, and short-acting opioids may be used to blunt sympathetic responses. Peripartum laryngeal edema may require smaller endotracheal tubes, which can limit bronchoscopic suctioning and should be anticipated. After 20 weeks, aortocaval compression in the supine position can reduce venous return; manual uterine displacement or left tilt should be maintained during induction and intubation. Immediate availability of vasopressors and vagolytics is essential, as abrupt hypotension and bradycardia may occur. Overall, peripartum intubation requires meticulous planning, experienced teams, appropriate equipment, and strict adherence to obstetric-adapted protocols [48].

## 16. Ventilatory Management in the Critically Ill Obstetric Patient

Ventilatory support in pregnancy follows general ICU principles but must also preserve uteroplacental gas exchange and prevent fetal acidosis. Consequently, levels of maternal hypoxemia or hypercapnia that might be tolerated in non-pregnant patients may be harmful when a viable fetus is present. Sustained maternal hypoxemia and prolonged hypercapnia impair placental diffusion, predisposing to fetal distress and combined respiratory/metabolic acidosis [49]. Indications for mechanical ventilation include oxygenation failure, ventilatory failure with CO_2_ retention, or unsustainable work of breathing. Although classic thresholds exist, decision-making in pregnancy should be more conservative, particularly with rapid deterioration, fatigue, altered consciousness, hemodynamic instability, or failure of non-invasive support [50].

When invasive ventilation is required, lung-protective ventilation is the cornerstone, especially in acute lung injury and ARDS, using low tidal volumes (~6 mL/kg predicted body weight) and maintaining plateau pressure generally <30 cmH_2_O to reduce volutrauma and barotrauma. PEEP should be titrated rationally and individualized, balancing oxygenation against hemodynamic tolerance; PEEP–FiO_2_ tables may assist. Blood gas targets require particular attention. In pregnant patients with ALI/ARDS, maternal PaCO_2_ is commonly maintained below 45–50 mmHg, avoiding both clinically significant hypercapnia and excessive hyperventilation. Marked hypocapnia may cause uteroplacental vasoconstriction and reduced fetal oxygen delivery. Targets should be individualized based on maternal disease severity, lung mechanics, hemodynamics, gestational age, and fetal viability.

Ventilator mode selection should prioritize patient–ventilator synchrony and airway pressure control, with vigilance for auto-PEEP and dynamic hyperinflation in tachypneic patients. Refractory ARDS may warrant advanced rescue strategies in experienced centers. Prone positioning deserves special emphasis: accumulated evidence—particularly from COVID-19-associated ARDS—supports that prone ventilation can be performed safely in selected pregnant patients, including in the second and third trimesters, when performed by trained teams with positioning that avoids abdominal and aortocaval compression. Proning may improve oxygenation and reduce injurious pressures, primarily benefiting the mother with indirect fetal benefit. Airway and aspiration risk, rapid desaturation, and positioning (including left uterine displacement when applicable) must be incorporated into intubation planning, consistent with peripartum airway challenges [51].

Whenever a viable fetus is present, fetal monitoring should be integrated when feasible, without superseding maternal stabilization. If severe maternal derangements persist despite optimized management, or if there is acute maternal–fetal deterioration, multidisciplinary discussion regarding delivery (including emergency cesarean preparedness and neonatal resuscitation) is required.

## 17. Point-of-Care Ultrasonography in Obstetric Critical Care

Point-of-care ultrasonography (POCUS) is increasingly central in modern critical care and is particularly valuable in the management of critically ill obstetric patients. It is dynamic, noninvasive, repeatable, and radiation-free, enabling rapid multisystem assessment to support time-sensitive decisions during maternal instability [52]. In obstetric critical care, POCUS should be viewed as an extension of the physical examination rather than a replacement for formal specialist imaging. It is especially useful in shock, acute respiratory failure, unexplained hemodynamic deterioration, oliguria, suspected thromboembolism, severe sepsis, and cardiac arrest [53].

Cardiovascular POCUS enables rapid estimation of left and right ventricular systolic function, chamber dilation, acute ventricular dysfunction, pericardial effusion, and indirect signs of tamponade. In shock, it supports differentiation among hypovolemic, distributive, cardiogenic, and obstructive etiologies, guiding fluids, vasopressors, and inotropes. Inferior vena cava assessment may contribute to evaluating fluid responsiveness but must be interpreted cautiously late in pregnancy due to aortocaval compression and physiological volume changes [54].

Lung ultrasound is particularly informative, as diffuse B-lines suggest interstitial pulmonary edema (e.g., severe preeclampsia, fluid overload, cardiac dysfunction); subpleural consolidations and dynamic air bronchograms support pneumonia; and absent pleural sliding suggests pneumothorax, especially relevant in mechanically ventilated patients. In acute respiratory failure, lung POCUS often outperforms chest radiography in sensitivity and can be repeated serially to monitor response [54].

Abdominal and renal POCUS supports evaluation of oliguria and acute kidney injury by identifying significant hydronephrosis, urinary retention, ascites, and indirect signs of hypovolemia. In selected scenarios, it may assist in identifying obstetric complications such as hemoperitoneum. Obstetric POCUS can also rapidly confirm fetal viability, presentation, amniotic fluid volume, and placental location when specialist assessment is unavailable, information that can be critical during maternal collapse and when considering emergency delivery.

During maternal cardiac arrest, POCUS may help identify reversible causes (e.g., tamponade, massive pulmonary embolism, profound hypovolemia) but must not interrupt or delay chest compressions. POCUS also guides invasive procedures (e.g., central venous access, pleural or abdominal drainage), improving safety and reducing complications, particularly in patients with coagulopathy or hemodynamic instability [55]. Effective use requires training and awareness of limitations, with interpretation contextualized to pregnancy physiology and integrated within multidisciplinary workflows. When appropriately implemented, POCUS supports more accurate diagnosis, safer interventions, and potentially improved maternal and fetal outcomes [56].

## 18. Vascular Access in Obstetric Critical Care

Reliable vascular access is essential in the critically ill obstetric patient for hemodynamic resuscitation, delivery of fluids, blood products, vasoactive agents, sedatives, antibiotics, and invasive monitoring. While general principles mirror those in non-pregnant patients, pregnancy-related anatomic, physiological, and hemostatic changes require careful planning and meticulous technique [57].

In emergencies, large-bore peripheral venous access should be obtained immediately—ideally two upper-limb cannulas (14 G or 16 G). Peripheral access remains first-line because it is rapid, safe, and sufficient for most early interventions, including massive transfusion. Lower-limb cannulation should be avoided, when possible, due to increased thromboembolic risk in pregnancy [58].

Central venous access is often required in shock, prolonged vasoactive infusions, difficult peripheral access, or when invasive monitoring is needed. In critically ill pregnant patients, the internal jugular vein is generally preferred because bleeding is more readily compressible. Subclavian access should be weighed cautiously—particularly in coagulopathy (e.g., massive obstetric hemorrhage, severe sepsis, HELLP)—given the risk of non-compressible bleeding. Femoral access, although rapid, should be reserved for selected situations because of higher infection and thrombosis risk and potential challenges in advanced gestation due to aortocaval compression [59].

Routine ultrasound guidance is strongly recommended for central access, improving success rates and reducing mechanical complications, especially in edema, obesity, distorted anatomy, or instability. Ultrasound also supports early detection of complications (e.g., pneumothorax) and may assist catheter position assessment.

Invasive arterial monitoring is indicated in hemodynamic instability, continuous blood pressure monitoring, vasoactive therapy, or frequent arterial blood gas sampling. The radial artery is commonly used due to accessibility and lower ischemic risk. Precise titration of vasoactive agents may help optimize uteroplacental perfusion when a viable fetus is present.

In refractory shock or circulatory failure, advanced access may be necessary (rapid infusion catheters, pressurized infusion systems, or extracorporeal cannulation in specialized centers). Decisions should be multidisciplinary, balancing maternal and fetal risks. Because pregnancy is hypercoagulable, catheter-associated thrombosis risk is increased; strict asepsis, daily reassessment of catheter necessity, and early removal are essential, with continuous reassessment of thrombotic versus hemorrhagic risk. Positioning matters: in advanced gestation, left uterine displacement or left lateral tilt should be used whenever feasible to reduce aortocaval compression and maintain venous return during procedures. Overall, safe and effective vascular access is integral to advanced life support in obstetric critical care and directly contributes to maternal stabilization and fetal protection [60].

## 19. Nutritional Support in the Critically Ill Obstetric Patient

Pregnancy entails progressively increased energy, protein, and micronutrient requirements. During critical illness, these needs coexist with stress-induced hypercatabolism (e.g., sepsis, trauma, organ failure), making nutritional support a core component of ICU management [61,62]. Even short periods of maternal undernutrition may lead to negative nitrogen balance, muscle loss, impaired immunity, delayed wound healing, and higher infection risk. In pregnancy, additional concerns include a propensity for ketosis during fasting, maternal metabolic acidosis, and reduced substrate delivery to the fetus. Thus, the goal is to meet maternal needs while preserving a stable fetal metabolic environment [63].

Enteral nutrition should be prioritized whenever feasible, given lower infection risk and maintenance of gut integrity. Early enteral feeding can be initiated cautiously even during mechanical ventilation, provided the patient is hemodynamically stable and has no absolute contraindications (e.g., ileus, bowel ischemia). However, delayed gastric emptying and aspiration risk are increased in pregnancy, requiring close monitoring, appropriate positioning, and post-pyloric tubes when needed. Because fasting ketosis can develop after relatively short periods, prolonged fasting should be avoided, and early carbohydrate provision is important to reduce lipolysis and ketone production—particularly in sepsis, respiratory failure, or severe preeclampsia. If enteral intake is not possible or insufficient, parenteral nutrition may be considered based on expected duration of gastrointestinal intolerance and overall maternal status, with strict monitoring of glucose, electrolytes, liver function, and catheter-related infection risk [64].

Caloric targets should avoid both underfeeding and overfeeding. Overfeeding increases hyperglycemia, hepatic lipogenesis, and CO_2_ production—especially relevant in respiratory failure. Adequate protein delivery supports lean mass and immune function and should be adjusted for renal and hepatic function. Metabolic monitoring is essential, with prompt correction of sodium, potassium, phosphate, and magnesium abnormalities. Glycemic control deserves special attention given physiological insulin resistance in pregnancy, often amplified by stress, corticosteroids, and sepsis; maternal hyperglycemia is associated with worse maternal and neonatal outcomes. Postpartum, energy demands may further increase with lactation, and nutrition plans should be adjusted accordingly. Overall, nutritional therapy should be treated as a therapeutic intervention integrated into multidisciplinary care.

## 20. Dialysis in the Critically Ill Pregnant Patient

The need for renal replacement therapy (RRT) in pregnancy is a marker of severe illness and is associated with increased maternal and perinatal morbidity and mortality. Acute kidney injury in pregnancy may arise from obstetric disorders or intercurrent medical conditions and requires coordinated care involving intensivists, obstetricians, nephrologists, and specialized nursing teams [65].

Indications for RRT are broadly similar to those in non-pregnant patients (refractory volume overload, severe/progressive hyperkalemia, persistent metabolic acidosis, symptomatic uremia, or clinical deterioration due to renal failure). In obstetrics, the initiation threshold is often lower because maternal metabolic and electrolyte derangements directly affect uteroplacental perfusion and fetal status [66]. Common obstetric causes include severe preeclampsia and complications (including HELLP), massive obstetric hemorrhage with hypovolemic shock, puerperal sepsis, amniotic fluid embolism, and disseminated intravascular coagulation. Non-obstetric causes include non-genital sepsis, glomerular diseases, systemic lupus erythematosus, thrombotic microangiopathy, and rhabdomyolysis [67].

Modality selection should reflect maternal hemodynamic stability, volume status, sepsis, institutional resources, and team expertise. Intermittent hemodialysis may be used in stable patients but can precipitate hypotension with adverse effects on uteroplacental blood flow. In unstable patients, continuous RRT (e.g., continuous veno-venous hemofiltration) is often preferred because it allows gradual fluid and solute control with fewer hemodynamic swings and may be safer for the fetus. Peritoneal dialysis is rarely used in advanced pregnancy due to mechanical limitations and infection risk [68,69].

During dialysis, preventing maternal hypotension is critical to avoid reduced placental perfusion and fetal distress. Ultrafiltration must be carefully controlled, with continuous blood pressure monitoring and appropriate prescription adjustments. Electrolyte correction—particularly sodium and potassium—should be gradual to avoid abrupt osmolar shifts that may affect the fetal compartment. Treating maternal acidosis is also important, as maternal acidosis can worsen fetal acid–base status. When a viable fetus is present, fetal surveillance before, during, and after sessions may be considered if maternal condition allows, but it must never delay maternal stabilization. Overall, RRT aims to restore maternal homeostasis and preserve uteroplacental perfusion [65].

## 21. Cardiopulmonary Bypass During Pregnancy

Cardiopulmonary bypass (CPB) in pregnancy is among the most complex scenarios in obstetric critical care, carrying substantial maternal and fetal risk. Nonetheless, in selected life-threatening conditions, it may be the only maternal life-saving option, consistent with the principle that fetal survival depends primarily on maternal stability [70]. Indications include catastrophic cardiac disease such as acute aortic dissection, critically decompensated valvular disease, unstable infective endocarditis, massive pulmonary embolism with circulatory collapse, and, rarely, advanced extracorporeal support for refractory cardiogenic shock. Decisions must be made by an experienced multidisciplinary team (cardiology, cardiac surgery, anesthesia, obstetrics, intensive care, neonatology) [69,71].

Physiologically, uteroplacental circulation lacks autoregulation and depends on maternal cardiac output, mean arterial pressure, and systemic vascular resistance. During CPB, hemodynamic changes, hemodilution, hypothermia, and systemic inflammation may reduce uteroplacental flow and precipitate fetal hypoxia, acidosis, and distress, contributing to historically high fetal loss rates—especially at earlier gestational ages [72]. Key technical principles to mitigate fetal risk include prioritizing normothermia when feasible, maintaining relatively high pump flow (often >2.5 L/min/m^2^), and targeting mean arterial pressure ≥ 70 mmHg to support uteroplacental perfusion. Adequate oxygenation and maintaining acceptable hematocrit are also essential. Fetal monitoring is desirable when technically feasible and gestational age allows meaningful interpretation, but it must not delay or compromise maternal intervention. Persistent fetal heart rate abnormalities most often reflect maternal hemodynamic compromise and should prompt optimization of maternal perfusion and oxygenation rather than isolated fetal interventions [73].

Gestational age strongly influences decision-making. In advanced gestation (approximately ≥28–32 weeks), delivery before CPB may be considered if maternal status allows minimal delay and delivery will not worsen instability. In earlier gestation, maternal stabilization is the overriding priority, accepting inherent fetal risk. There is no universal consensus on timing of delivery relative to CPB; decisions are individualized [74].

Post-CPB care requires ICU monitoring, organ support as needed, and obstetric reassessment, with attention to bleeding risk given CPB-related coagulopathy. Contemporary reports suggest improving maternal outcomes in specialized centers, with declining fetal morbidity as perfusion strategies become more physiological.

## 22. Extracorporeal Membrane Oxygenation in the Critically Ill Pregnant Patient

Extracorporeal membrane oxygenation (ECMO) has become an important rescue strategy for pregnant patients with severe, refractory cardiorespiratory failure. It is a temporary support modality to allow maternal pulmonary and/or cardiac recovery, not definitive therapy [75]. In obstetrics, the most common indication is refractory hypoxemic respiratory failure, particularly severe ARDS despite lung-protective ventilation, optimized positive end-expiratory pressure (PEEP), recruitment strategies, and prone positioning. Less frequent indications include severe cardiogenic shock, massive pulmonary embolism, and cardiovascular collapse related to cardiomyopathies [42]. VV-ECMO is preferred for isolated respiratory failure, whereas VA-ECMO is reserved for significant circulatory failure or refractory cardiac arrest. Pregnancy is not an absolute contraindication, but ECMO implementation requires careful risk assessment—especially bleeding, thrombosis, and anticoagulation-related complications. Obstetric coagulopathy, active hemorrhage, or high bleeding risk increases complexity and mandates intensive multidisciplinary oversight.

Timing of delivery in patients on ECMO is a major dilemma. When possible, maternal stabilization should precede delivery, with decisions individualized by gestational age, fetal viability, and maternal physiology. In some cases, delivery may improve respiratory mechanics and hemodynamics; in others, continuing pregnancy until greater stability is preferable. No fixed rule exists, and decisions require coordinated input from intensivists, obstetricians, anesthesiologists, neonatologists, and ECMO specialists.

Fetal survival depends primarily on maternal stability and gestational age. Continuous fetal monitoring may be considered when a viable fetus is present and logistics allow, but it must not interfere with maternal support priorities, reinforcing the principle that optimal fetal care is effective maternal stabilization [25]. Overall, ECMO should be considered early in experienced centers—before irreversible organ injury—and its availability underscores the importance of well-organized referral systems, clear protocols, and integrated multidisciplinary care [42].

## 23. Blood Component Replacement and Viscoelastic Coagulation Assessment in Obstetric Critical Care

Blood component therapy is a cornerstone of obstetric critical care and is frequently required in massive obstetric hemorrhage, acquired coagulopathies, severe sepsis, HELLP syndrome, disseminated intravascular coagulation, and pregnancy-associated hematologic disorders. Unlike isolated transfusion, goal-directed component replacement aims to restore oxygen-carrying capacity, effective hemostasis, and intravascular volume while minimizing maternal complications and fetal repercussions [76].

Obstetric bleeding may be sudden and profound, rapidly exceeding physiological compensation. Crystalloids or colloids alone are inadequate and may worsen hemodilution, coagulopathy, and tissue hypoxia. Contemporary management, therefore, emphasizes early, balanced blood component administration guided by clinical status, laboratory testing, and—when available—viscoelastic assays [77].

Packed red blood cells are used to restore oxygen delivery. A rigid hemoglobin threshold is not appropriate in critically ill obstetric patients; although transfusion is often reasonable below 7 g/dL in stable patients, higher targets may be needed with active hemorrhage, shock, hypoxemia, cardiovascular dysfunction, or fetal compromise, recognizing that severe maternal anemia directly reduces fetal oxygen delivery. Fresh frozen plasma is indicated to correct multiple coagulation factor deficiencies, particularly with active bleeding or before urgent invasive procedures. Empiric plasma may be necessary when clinical coagulopathy is evident and laboratory confirmation is delayed.

Thrombocytopenia is common in critical obstetrics, and platelets are indicated with active bleeding, surgery, or unsafe platelet counts. In practice, transfusion is typically considered when platelets are <50,000/mm^3^ in hemorrhagic or operative settings, while lower thresholds may be acceptable in stable, non-bleeding patients. Fibrinogen requires particular attention: in pregnant patients with bleeding, levels < 200 mg/dL are associated with progression to severe hemorrhage. Early replacement with cryoprecipitate or fibrinogen concentrate is recommended when fibrinogen falls, especially in placental abruption, amniotic fluid embolism, and severe uterine atony. When massive hemorrhage occurs, early activation of institutional massive transfusion protocols is essential. These commonly deliver red cells, plasma, and platelets in a balanced approach (often approximating 1:1:1) alongside definitive source control and advanced hemodynamic support. Serial laboratory testing (hemoglobin, PT/INR, aPTT, fibrinogen, platelet count) should guide ongoing therapy [76].

Viscoelastic tests such as thromboelastography (TEG) or rotational thromboelastometry (ROTEM) provide real-time, global assessment of clot formation, strength, and lysis, enabling more targeted therapy than conventional tests—particularly relevant in pregnancy, a physiologically hypercoagulable state in which isolated PT/INR/aPTT may be misleading. Typical patterns include prolonged reaction time suggesting factor deficiency (supporting plasma), reduced alpha angle or maximum amplitude suggesting fibrinogen deficiency and/or platelet dysfunction (supporting fibrinogen/cryoprecipitate and/or platelets), and early clot lysis indicating hyperfibrinolysis (supporting antifibrinolytics such as tranexamic acid). In severe obstetric hemorrhage, viscoelastic-guided protocols can reduce empiric transfusion and limit complications of massive transfusion without clear signals of increased thrombosis, while also supporting decision-making in sepsis, acute liver dysfunction, severe preeclampsia, and HELLP. Overall, component replacement should be early, balanced, goal-directed, and integrated with treatment of the underlying cause, as correcting anemia, coagulopathy, and hypovolemia is decisive for maternal and fetal survival [78].

## 24. Maternal and Fetal Monitoring in the Obstetric ICU

Monitoring in the obstetric ICU should be tailored to maternal severity, organ support needs, and ongoing fetal assessment. Patients requiring moderate-to-advanced support often benefit from invasive monitoring, as clinical assessment alone may miss early physiological deterioration [37].

Invasive arterial blood pressure monitoring is frequently essential in critically ill pregnant patients, enabling continuous perfusion assessment, precise titration of fluids and vasopressors, and repeated arterial blood gas analysis. Central venous access is commonly used; internal jugular placement is generally favored for safety, but central venous pressure (CVP) should be interpreted cautiously antepartum—especially after ~24–26 weeks—because aortocaval compression can artifactually elevate readings. Similarly, inferior vena cava ultrasound is less reliable in late pregnancy; bedside transthoracic echocardiography is often more informative for integrated hemodynamic assessment (cardiac output surrogates, ventricular filling, contractility, structural dysfunction) [79].

Continuous cardiac output monitoring technologies are increasingly available, but derived indices (e.g., pulse pressure or stroke volume variation) may be more useful postpartum than antepartum because late-gestation aortocaval compression alters preload and dynamic indices. Fetal status should be integrated into ICU care because it reflects maternal oxygenation, perfusion, acid–base balance, and medication effects. Surveillance should be individualized according to gestational age, fetal viability, maternal condition, and goals of care. Options range from intermittent Doppler auscultation to continuous cardiotocography (CTG), biophysical profile (BP), and maternal–fetal Doppler assessment. CTG is commonly used in viable pregnancies when maternal status permits, but non-reassuring patterns may reflect maternal hypoxemia, acidosis, hemodynamic instability, or sedative exposure; persistent abnormalities—particularly with maternal deterioration—may influence delivery decisions [25]. Doppler assessment provides hemodynamic insight (uterine, umbilical, middle cerebral artery, ductus venosus) and can detect progressive placental and fetal compromise, but no single test should trigger automatic intervention. Fetal monitoring should support multidisciplinary decision-making while reaffirming that maternal stabilization is the primary determinant of fetal outcome.

## 25. Delivery in the Critically Ill Pregnant Patient

Decisions regarding timing and mode of delivery are among the most complex elements of obstetric critical care and should generally follow initial maternal stabilization. Once stabilization is achieved, fetal assessment is undertaken, consistent with the principle that maternal resuscitation and support remain the priority because fetal prognosis depends on maternal physiology [48,80].

Management requires a multidisciplinary team (intensive care, obstetrics, maternal–fetal medicine, obstetric anesthesia, neonatology, and other specialists as indicated) and early, transparent communication with the family. In high-risk scenarios, resources for urgent delivery should be immediately available in the ICU setting [28].

In pregnancies beyond 22–24 weeks, antenatal corticosteroids for fetal lung maturation should be considered when time and maternal condition permit. Tocolysis requires careful risk–benefit assessment, particularly in critically ill patients, and beta-agonists should generally be avoided. If imminent preterm delivery is expected (especially <32 weeks), magnesium sulfate for fetal neuroprotection may be considered if not contraindicated.

Mode of delivery should be individualized and driven primarily by maternal safety, fetal viability, and response to ICU therapy. Anesthetic choice (general vs. neuraxial) depends on coagulation status, airway risk, and hemodynamic stability. Because uteroplacental blood flow depends largely on maternal mean arterial pressure, maternal hypotension is a major modifiable threat; the combination of hypotension, hypoxemia, and anemia is particularly harmful to fetal oxygen delivery. Maintaining adequate maternal hemoglobin (often ≥7 g/dL, individualized) and correcting coagulopathy before operative or vaginal delivery are key principles [81].

Selected scenarios warrant evaluation of fetomaternal hemorrhage (e.g., Rh-negative trauma), using Kleihauer–Betke testing and anti-D immunoglobulin when indicated; prophylaxis may also be considered after Rh-untested platelet transfusion in Rh-negative patients.

ICU admission should not preclude good peripartum practices when feasible. With appropriate safeguards, selected humanization measures may be incorporated, including the presence of a support person, skin-to-skin contact, and individualized umbilical cord clamping decisions. Immediate clamping may be necessary with severe maternal instability, active hemorrhage, urgent maternal resuscitation, critically impaired uteroplacental perfusion, or when neonatal resuscitation is required and bedside resuscitation is not feasible. Lactation support should be encouraged early when possible, including milk expression during temporary separation. Implementing these practices requires planning, cross-team coordination, and continuous reassessment; humanized care is compatible with safety when adapted to clinical constraints.

## 26. Cardiopulmonary Resuscitation in Pregnancy

Maternal cardiac arrest in pregnancy is rare but catastrophic and requires immediate, coordinated response with pregnancy-specific modifications. Standard advanced life support principles apply, but adaptations are essential to optimize maternal resuscitation and thereby fetal survival. Restoration of maternal circulation remains the overriding priority [82].

High-quality chest compressions should begin immediately (100–120/min, depth ~5–6 cm). From ≥20 weeks’ gestation, continuous manual left uterine displacement is mandatory to relieve aortocaval compression and improve venous return and cardiac output. Hand position for compressions may need to be slightly more cephalad due to diaphragmatic elevation [83].

Ventilation should be delivered with 100% oxygen using a 30:2 compression–ventilation ratio until an advanced airway is placed. After intubation, compressions continue uninterrupted with ventilation about one breath every 6 s, avoiding hyperventilation. Early intubation by an experienced operator is recommended because pregnancy increases difficult airway and aspiration risk [84]. Defibrillation uses standard adult energy levels, and resuscitation medications follow standard algorithms (e.g., epinephrine 1 mg IV every 3–5 min; amiodarone for refractory VF/pulseless VT; magnesium sulfate in selected contexts such as eclampsia or torsades). Teams should aggressively identify and treat reversible causes, with heightened attention to obstetric etiologies (massive hemorrhage, pulmonary embolism, sepsis, eclampsia, peripartum cardiomyopathy, high neuraxial block, amniotic fluid embolism) [85].

If there is no return of spontaneous circulation after ~4 min of effective CPR in pregnancies ≥ 20–24 weeks, resuscitative perimortem cesarean delivery should be initiated immediately, aiming for delivery by 5 min. The primary purpose is to improve maternal resuscitation by relieving aortocaval compression and reducing metabolic demand; potential fetal survival is secondary and depends on gestational age. After ROSC, patients require advanced ICU care, etiologic workup, and multidisciplinary planning regarding continuation or delivery of the pregnancy [83].

## 27. Vasopressors and Hemodynamic Support in Critically Ill Pregnant and Postpartum Patients

Vasopressors play a central role in the management of circulatory failure in critically ill pregnant and postpartum patients. Although physiologic pregnancy is characterized by reduced systemic vascular resistance and increased cardiac output, shock states in pregnancy follow the same fundamental pathophysiologic principles observed in non-pregnant patients. Importantly, vasopressors should not be withheld when clinically indicated, as restoration of adequate maternal perfusion pressure is essential to maintain uteroplacental blood flow and fetal oxygen delivery [1].

Initial hemodynamic management should prioritize early recognition of shock phenotype, integrating clinical examination, laboratory markers, and bedside monitoring. Fluid resuscitation must be individualized, as pregnant patients are particularly susceptible to fluid overload due to reduced colloid oncotic pressure, increased capillary permeability in critical illness, and rapid intravascular–extravascular fluid shifts. When hypotension persists despite cautious fluid administration, early initiation of vasopressor therapy is recommended to avoid prolonged maternal hypoperfusion [2].

Mean arterial pressure (MAP) targets should be tailored to the individual patient, taking into account baseline blood pressure, gestational age, comorbidities, and evidence of end-organ perfusion. While no pregnancy-specific MAP threshold has been universally established, maintaining adequate maternal perfusion remains the primary therapeutic objective, as fetal well-being is directly dependent on maternal hemodynamic stability rather than on uterine-specific targets [13].

Bedside echocardiography and POCUS are valuable adjuncts in guiding vasopressor therapy, allowing rapid assessment of cardiac function, volume status, and shock etiology. These tools support dynamic titration of vasoactive agents and help distinguish distributive, cardiogenic, and mixed shock states, which are not uncommon in obstetric critical illness [24].

In the presence of a viable fetus, maternal–fetal considerations should inform, but not delay, hemodynamic intervention. Current evidence supports the principle that optimizing maternal circulation is the most effective strategy to preserve fetal perfusion, and delays in vasopressor initiation may worsen outcomes for both mother and fetus. Postpartum patients require particular vigilance, as abrupt physiological changes following delivery may unmask or exacerbate hemodynamic instability.

## 28. Perimortem Cesarean Delivery (Resuscitative Hysterotomy) Considerations

Perimortem (resuscitative) cesarean delivery is an extreme but potentially life-saving intervention for refractory maternal cardiac arrest. Its primary goal is maternal resuscitation—improving venous return and cardiac output via aortocaval decompression and reducing maternal metabolic demand—while fetal benefit is secondary [86]. It is indicated when ROSC has not occurred after ~4 min of effective CPR in pregnancies ≥ 20–24 weeks (or when the uterine fundus is at/above the umbilicus). The decision should be clinical and time-based and must not await ultrasound confirmation of fetal viability or gestational age. It should be performed where resuscitation is occurring (ICU, ED, or OR) without interrupting chest compressions; transfer should not delay the procedure [87]. Preparation must be minimal, prioritizing speed over extensive sterile setup, given the time-dependent nature of benefit [88,89].

A midline infraumbilical laparotomy provides rapid access, followed by uterine entry (as exposure allows) and immediate fetal delivery. The cord is clamped promptly and neonatal care proceeds as indicated. Following uterine evacuation, CPR effectiveness and hemodynamics may improve. If ROSC occurs, rapid uterine hemostasis should be achieved using the most expedient measures available. Importantly, this is an advanced resuscitation procedure rather than a purely obstetric operation; lack of an obstetrician should not delay action when trained clinicians are present. Early recognition, rapid execution, and integrated team response are decisive for maternal—and potentially fetal—outcomes [88].

## 29. Safe Use of Radiation in Pregnancy

Imaging that uses ionizing radiation often raises concern in pregnancy, particularly in emergency and ICU settings. However, radiological studies may be essential for the timely diagnosis and management of life-threatening conditions. In these circumstances, maternal health must be prioritized because diagnostic or therapeutic delay generally poses far greater fetal risk than appropriately performed, dose-optimized imaging [90].

Fetal radiosensitivity varies by gestational age. During the pre-implantation period, exposure follows an “all-or-none” pattern (possible embryonic loss without increased malformation risk). During organogenesis—especially weeks 2–8—teratogenic risk is theoretically higher, but deterministic effects occur only at doses substantially above those used in most diagnostic examinations. In the second and third trimesters, malformation risk is minimal; the main theoretical concerns are growth effects and a small stochastic increase in childhood cancer risk, still extremely low at typical diagnostic doses [91].

In general, fetal doses < 50 mGy are not associated with proven increases in congenital malformations, growth restriction, intellectual disability, or pregnancy loss. Most commonly used studies—such as chest radiography, head computed tomography (CT), and chest CT—deliver fetal doses well below this threshold. Even higher-exposure examinations (e.g., abdominopelvic CT) rarely reach deterministic-effect ranges when appropriately performed [92].

In critically ill pregnant patients, imaging should be driven by clear clinical indications and a structured risk–benefit assessment (Table 6). If an examination is necessary for diagnosis or therapeutic decision-making, it should not be delayed or replaced by inferior alternatives when that compromises maternal care. When feasible and diagnostically adequate, non-ionizing modalities (ultrasound and magnetic resonance imaging—MRI) are preferred [93].

When ionizing radiation is required, exposure should be minimized using As Low As Reasonably Achievable (ALARA) principles: lowest dose compatible with diagnostic quality, appropriate collimation, limiting the field of view, and abdominal shielding when technically feasible (particularly for non-abdominal studies). Iodinated contrast CT can be used when clinically indicated; although iodine crosses the placenta, consistent teratogenic effects have not been demonstrated. Neonatal thyroid function assessment may be considered after substantial iodinated contrast exposure. Gadolinium contrast for MRI should generally be avoided except in exceptional circumstances due to associations with adverse fetal outcomes in observational data. Clear communication with the patient and family—addressing often overestimated risks—supports shared decision-making, and documentation of indication and counseling is advisable, especially in emergencies. Overall, radiation use in pregnancy is safe when clinically justified, technically optimized, and focused on maternal stabilization as the central determinant of fetal outcome [94].

## 30. Why Multidisciplinary and Multiprofessional Care Matters in Obstetric Critical Care

Caring for the critically ill pregnant patient is among the most complex scenarios in modern medicine, requiring integrated decisions that affect two physiologically interdependent entities: mother and fetus. Fragmented care is insufficient; outcomes depend on coordinated, interdisciplinary practice [95].

The obstetrician remains central for pregnancy assessment, delivery timing and route, and fetal implications of maternal disease. The intensivist provides systematic management of organ dysfunction and coordinates hemodynamic, ventilatory, and metabolic support in the ICU. The obstetric anesthesiologist is essential for airway management, analgesia, and anesthesia for urgent procedures, including operative delivery and resuscitative interventions.

Early involvement of other medical specialists is often required. Hematology is critical for complex coagulopathies, massive hemorrhage, and interpretation of viscoelastic testing (e.g., TEG) to enable more targeted hemostatic therapy. Infectious disease expertise supports sepsis diagnosis, antimicrobial selection, and source control—particularly with multidrug resistance. Surgical teams (general, vascular, thoracic) may be needed for trauma, acute abdomen, non-obstetric bleeding, or surgical complications.

Multiprofessional care is equally indispensable. Physiotherapists are key to ventilatory management, pulmonary complication prevention, early mobilization, and ventilator weaning—especially important given reduced maternal respiratory reserve. Speech-language therapy supports swallowing and airway protection after prolonged intubation. Psychology helps patients and families cope with critical illness, prognostic uncertainty, and ethically difficult decisions. Specialized ICU/obstetric nursing provides continuous surveillance, protocol implementation, safe therapy delivery, and effective communication across teams; social workers support family needs and logistics. Collaborative models with structured communication and clear protocols are consistently linked to safer care and better maternal–perinatal outcomes. Obstetric critical care is therefore intrinsically interdisciplinary, and excellence depends on integration, not a single specialty [96,97].

## 31. Post-Intensive Care Syndrome and Maternal Mental Health in Obstetric Critical Care

Survival from critical illness does not mark the end of morbidity for many obstetric patients. Post-intensive care syndrome (PICS), a constellation of physical, cognitive, and psychological sequelae following ICU admission, represents an increasingly recognized outcome in maternal critical care and has unique implications for maternal–infant health. In pregnant and postpartum women, PICS may profoundly affect recovery, parenting capacity, and long-term well-being [1].

Psychological manifestations are particularly relevant in this population. Anxiety, depression, post-traumatic stress disorder (PTSD), and sleep disturbances are common after ICU admission and may be exacerbated by emergency delivery, loss of perceived bodily control, and fear for fetal or neonatal survival. Mother–infant separation, frequently unavoidable during critical illness, further increases the risk of postpartum depression, bonding disorders, and impaired maternal–infant attachment. These effects may persist well beyond hospital discharge, influencing both maternal mental health and child development [1].

Physical and cognitive components of PICS, including weakness, fatigue, impaired concentration, and memory deficits, may interfere with breastfeeding, newborn care, and reintegration into daily life. The postpartum period, already characterized by physiologic stress and psychosocial vulnerability, may amplify these sequelae. Importantly, traditional ICU outcome measures focused solely on mortality fail to capture these longer-term, patient-centered consequences [1].

Mitigation of PICS in obstetric critical care should begin during the ICU stay. Practical strategies include promoting family presence whenever feasible, facilitating early and structured communication with partners and family members, and supporting lactation through milk expression and preservation when direct breastfeeding is not possible. Early mobilization, delirium prevention bundles, adequate analgesia and sedation practices, and attention to sleep hygiene are essential components of PICS prevention. When separation from the newborn is unavoidable, intentional efforts to maintain maternal, infant connection, such as photographs, video contact, or updates from neonatal teams, may help preserve bonding [98].

Post-ICU care is equally critical. Early identification of psychological distress and structured follow-up with mental health screening for depression, anxiety, and PTSD should be integrated into postpartum care pathways for women recovering from critical illness. Multidisciplinary collaboration among intensive care, obstetrics, mental health professionals, and primary care is essential to address the complex needs of this population [99].

In obstetric critical care, successful outcomes should be defined not only by survival, but also by functional recovery, psychological health, and the preservation of maternal–infant relationships. Recognition of PICS and implementation of preventive and supportive strategies are therefore integral components of high-quality maternal intensive care [99].

## 32. Expanding Obstetric Critical Care: Tele-ICU, Artificial Intelligence, and New Care Models

Improving access to specialized obstetric critical care is challenging, particularly where regional inequities limit specialist availability. Tele-ICU models offer a promising strategy to extend expertise from tertiary centers to hospitals with limited intensivist or maternal–fetal medicine coverage, potentially improving outcomes for critically ill obstetric patients [65].

Tele-ICUs enable continuous or on-demand remote consultation, including shared review of vital signs, labs, imaging, ventilator settings, and complex therapeutic decisions. In obstetric critical care—where maternal–fetal tradeoffs are nuanced—timely expert input may be decisive [98]. In general ICU populations, tele-ICU programs have been associated with reduced mortality, shorter ICU stay, fewer complications, and more efficient resource use, although results vary by system integration and organizational design. Obstetric-specific evidence remains limited, but similar benefits are plausible, particularly in low-volume or under-resourced settings.

Tele-ICU support can assist with ICU transfer decisions, ARDS ventilatory strategy, sepsis and shock management, complex hemodynamic interpretation, fetal monitoring strategy, and planning delivery timing and route. It may also facilitate shared decisions in rare, high-stakes scenarios (e.g., resuscitative cesarean delivery or extracorporeal support). However, tele-ICU does not replace trained local teams or minimal infrastructure; effectiveness depends on clear communication, defined responsibilities, protocol integration, and institutional acceptance. Limitations include technological dependence, implementation costs, and legal/ethical considerations [99,100].

In parallel, artificial intelligence (AI) is increasingly used in critical care to detect early deterioration and predict sepsis, shock, respiratory failure, mechanical ventilation, or ICU admission needs. In obstetrics, AI may be particularly valuable when integrated with early warning systems (e.g., MEOWS), improving sensitivity before overt collapse. AI can also identify patterns across high-volume physiologic and laboratory data that are difficult to detect clinically, supporting triage and bed allocation in tele-ICU networks—always under clinician oversight. Importantly, AI is an adjunct rather than a substitute: obstetric critical care decisions also involve patient values, fetal viability, and ethical/legal considerations, requiring transparent and obstetric-validated tools embedded within robust protocols.

Despite their potential, both Tele-ICU and artificial intelligence–based tools present important limitations that warrant explicit consideration in obstetric critical care. Tele-ICU effectiveness is highly dependent on the availability of reliable technological infrastructure and adequately trained bedside teams; remote expertise cannot compensate for deficiencies in local staffing, monitoring, or fundamental critical care capabilities. Integration into clinical workflows may be challenging, particularly when roles, accountability, and escalation pathways are not clearly defined, potentially leading to delays rather than improvements in care. Furthermore, legal and ethical issues, including data security, cross-institutional responsibility, and medico-legal accountability for remote decision-making, remain incompletely resolved in many health systems [99].

AI-driven systems face additional constraints, especially in obstetric populations. Many algorithms are trained on non-pregnant cohorts and lack obstetric-specific validation, raising the risk of misclassification when physiological adaptations of pregnancy (such as baseline tachycardia, leukocytosis, or altered blood pressure thresholds) are interpreted as pathologic, or conversely when true clinical deterioration is overlooked due to the high compensatory reserve of pregnancy. Algorithm bias, limited generalizability, calibration drift, and false-positive alerts may contribute to alarm fatigue and reduced clinician trust. Importantly, the interpretability of AI outputs is critical in obstetric care, where decisions frequently involve maternal–fetal tradeoffs, ethical considerations, and patient values [100].

For these reasons, Tele-ICU and AI should be regarded as adjunctive decision-support tools, operating under continuous clinician oversight, rather than as substitutes for experienced multidisciplinary teams and well-structured obstetric critical care systems. Overall, tele-ICU and AI may reduce inequities, improve consistency, and support better maternal–perinatal outcomes when integrated with strong local care and referral systems [98,99,100].

## 33. Ethical Dilemmas in Obstetric Critical Care

Obstetric critical care is ethically distinctive because decisions often involve two interdependent patients in time-pressured, high-uncertainty contexts. Rarely, continuing pregnancy may pose imminent, non-reversible maternal risk despite optimal support (e.g., refractory cardiac failure, severe pulmonary hypertension, progressive multiorgan failure). In such situations, pregnancy termination may be the only life-saving option for the woman, grounded in beneficence and non-maleficence and supported—when applicable—by national legal frameworks permitting termination to preserve maternal life. These decisions require careful clinical assessment, thorough documentation, and, whenever possible, multidisciplinary discussion and transparent communication with the patient and family [101].

The core ethical principle is primacy of maternal life and autonomy: the mother is the only fully autonomous patient, able to express preferences and values. Maternal diagnostic and therapeutic interventions should not be withheld solely due to fetal risk, particularly when omission endangers both [102].

A frequent dilemma concerns delivery timing. In some obstetric conditions (e.g., severe preeclampsia/HELLP), delivery is part of maternal treatment. In many non-obstetric illnesses, early delivery rarely alters maternal disease trajectory and may expose the neonate to severe prematurity risks. Decisions must balance beneficence and non-maleficence, integrating gestational age, fetal viability, maternal severity, and response to therapy [103].

Advanced life support technologies (prolonged ventilation, RRT, ECMO) introduce further tension when maternal prognosis is uncertain: support may be continued to gain fetal maturity, but may also approach futility. These decisions require explicit clinical criteria, periodic reassessment, and candid family discussions aligned with the patient’s known values.

Resuscitation in pregnancy and the need for resuscitative cesarean delivery are extreme time-critical scenarios where consent is often impossible; beneficence and standardized protocols guide action in the presumed best interest of mother and fetus. Fetal monitoring can also create pressure toward urgent delivery; teams must recognize that fetal compromise in the ICU often reflects maternal deterioration, and inappropriate fetal prioritization can harm both. Ethical care therefore depends on clear communication, multidisciplinary deliberation, and institutional protocols that support consistent, accountable decision-making centered on the woman’s dignity and her family [104,105].

## Figures and Tables

**Table 1:** Pregnancy-related maternal physiological changes relevant to critical illness and ICU management.

System	Physiological Adaptation in Pregnancy	Implications for Critical Illness and ICU Care
Cardiovascular	↑ Cardiac output (30–50%); ↓ systemic vascular resistance; mid-gestation ↓ MAP	Shock may be masked until late; significant blood loss may occur before hypotension; MAP targets must be individualized
	Aortocaval compression after ~20 weeks	Reduced venous return in supine position; need for left uterine displacement during resuscitation and procedures
Respiratory	↓ Functional residual capacity; ↑ oxygen consumption; chronic respiratory alkalosis	Rapid desaturation during apnea or respiratory failure; limited respiratory reserve; cautious use of permissive hypercapnia
Hematologic	Physiologic anemia; hypercoagulable state	Reduced oxygen-carrying reserve; ↑ risk of thromboembolism, especially with immobility, sepsis, or postpartum
Renal	↑ Renal plasma flow and GFR; ↓ baseline creatinine	“Normal” non-pregnant creatinine may indicate AKI; ↑ clearance of renally excreted drugs
Hepatic	Altered liver enzyme levels; ↓ albumin	Interpretation of transaminases may be misleading; ↑ free fraction of protein-bound drugs
Gastrointestinal	↓ Lower esophageal sphincter tone; delayed gastric emptying	High aspiration risk; all critically ill pregnant patients treated as full stomach
Endocrine/Metabolic	Insulin resistance; ↑ lipolysis; rapid ketosis with fasting	Higher risk of metabolic derangements; avoid prolonged fasting; early nutritional support essential
Immunologic	Modulated immune response	Increased susceptibility to severe infection and atypical sepsis presentation
Postpartum physiology	Autotransfusion; mobilization of extravascular fluid	High risk of acute decompensation (heart failure, pulmonary edema, hypertension) despite apparent stability

Abbreviations: MAP, mean arterial pressure; GFR, glomerular filtration rate; AKI, acute kidney injury.

**Table 2:** Common diagnostic pitfalls in obstetric critical care and practical ICU implications.

Physiological Change	Potential Misinterpretation	Clinical Risk	Practical ICU Implication
Physiologic tachycardia	Interpreted as anxiety or pain	Delayed recognition of hemorrhage or sepsis	Persistent tachycardia requires investigation even with normal BP
Leukocytosis of pregnancy	Assumed infection	Unnecessary antibiotics or missed true infection	Interpret trends and clinical context rather than isolated values
Low baseline creatinine	Considered normal renal function	Missed early AKI	Small creatinine rises may represent significant renal injury
Mild respiratory alkalosis	Viewed as hyperventilation	Inappropriate ventilation targets	Respect pregnancy-adapted PaCO_2_ goals
Preserved BP in shock	“Hemodynamic stability”	Delayed shock treatment	Evaluate perfusion markers (lactate, urine output, mental status)
Dyspnea in pregnancy	Attributed to physiologic changes	Missed PE, pulmonary edema, ARDS	New or progressive dyspnea warrants urgent evaluation
Postpartum stability	Assumed resolution of risk	Delayed ICU referral	Immediate postpartum period is highest-risk window
Non-pregnant sepsis criteria	Over- or under-classification	Delayed or inappropriate escalation	Use obstetric-adjusted tools (MEOWS, SOS)

Abbreviations: BP, blood pressure; AKI, acute kidney injury; ARDS, acute respiratory distress syndrome; ICU, intensive care unit; PaCO_2_, partial pressure of arterial carbon dioxide; MEOWS, Modified Early Obstetric Warning System; SOS, Sepsis in Obstetrics Score.

**Table 3:** Modified Early Obstetric Warning Score (MEOWS).

Parameter	Normal	Yellow Alert	Red Alert
Heart rate (bpm)	60–100	101–120 or 50–59	>120 or <50
Systolic blood pressure (mmHg)	100–139	90–99 or 140–159	<90 or ≥160
Diastolic blood pressure (mmHg)	60–89	90–99	≥100
Respiratory rate (breaths/min)	12–20	21–30	>30 or <10
Temperature (°C)	36.0–37.4	37.5–38.0 or 35.5–35.9	>38.0 or <35.5
Oxygen saturation (%)	≥96	94–95	<94
Level of consciousness	Alert	Responds to voice	Responds to pain or unresponsive
Urine output (mL/h)	≥30	20–29	<20

Data derived from [24].

**Table 4:** Clinical Presentation of Conditions Most Commonly Seen in Critically Ill Obstetric Patients.

Obstetric Causes	Pre-Existing Diseases That May Worsen During Pregnancy	Conditions with Increased Susceptibility During Pregnancy
Hypertensive crisis Severe preeclampsia Magnesium toxicity HELLP syndrome Eclampsia Placental abruption Uterine rupture Uterine inversion Retained products of conception Placenta accreta spectrum Acute fatty liver of pregnancy Obstetric hemorrhage Ruptured ectopic pregnancy Obstetric sepsis (chorioamnionitis) Septic abortion Endometritis Ovarian hyperstimulation syndrome Amniotic fluid embolism Peripartum cardiomyopathy Tocolytic-induced pulmonary edema/heart failure	**Cardiovascular:** Valvular heart disease Aortic coarctation Chronic (systemic) hypertension Congenital heart disease Coronary artery disease Pulmonary hypertension Marfan syndrome (aortic root > 4 cm) Post–heart transplantation **Respiratory:** Post–lung transplantation Cystic fibrosis **Renal:** Chronic kidney disease **Endocrine:** Prolactinomas Diabetic ketoacidosis **Connective tissue diseases:** Scleroderma Polymyositis Systemic lupus erythematosus **Neurologic:** Epilepsy Intracranial neoplasms **Hepatic:** Cirrhosis Budd–Chiari syndrome **Hematologic:** Sickle cell disease	**Renal:** Acute kidney injury **Infectious:** Urosepsis Hepatitis E Varicella pneumonia Falciparum malaria Influenza (H1N1 and other strains) **Hematologic:** Disseminated intravascular coagulation Hemolytic uremic syndrome Thrombotic thrombocytopenic purpura Deep vein thrombosis **Endocrine:** Gestational diabetes Sheehan syndrome **Central nervous system:** Intracranial hemorrhage Cerebral venous thrombosis **Respiratory:** Pulmonary embolism Air embolism Pulmonary aspiration

Data derived from [25].

**Table 5:** Sequential Organ Failure Assessment (SOFA) Score.

System/Score	0	1	2	3	4	Clinical Notes
Respiratory (PaO_2_/FiO_2_)	≥400	<400	<300	<200 (MV)	<100 (MV)	Ventilatory support
Coagulation (Platelets × 10^3^/mm^3^)	≥150	<150	<100	<50	<20	Bleeding risk
Liver (Bilirubin, mg/dL)	<1.2	1.2–1.9	2.0–5.9	6.0–11.9	≥12.0	Assess obstetric etiologies
Cardiovascular	MAP ≥ 70	MAP < 70	Dopamine ≤ 5/Dobutamine	Dopamine 5–15/Norepi ≤ 0.1	Dopamine > 15/Norepi > 0.1	Vasopressor use
Central nervous system (Glasgow Coma Scale)	15	13–14	10–12	6–9	<6	Neurologic impairment
Renal (Creatinine or urine output)	<1.2	1.2–1.9	2.0–3.4	3.5–4.9 or <500 mL/day	≥5.0 or <200 mL/day	Consider dialysis

Abbreviations: MV, mechanical ventilation; MAP, mean arterial pressure; Norepi, norepinephrine. Data derived from [31].

**Table 6:** Estimated Fetal Exposure to Ionizing Radiation from Common Radiologic Examinations During Pregnancy (Adapted from ACOG, 2017).

Radiologic Examination	Estimated Fetal Dose (mGy)	Notes
Chest radiograph—two views	<0.005–0.01	Very low-dose exam (<0.1 mGy)
Spine radiograph—AP and lateral	<0.001	Very low-dose exam (<0.1 mGy)
Mammography—two views	0.001–0.01	Very low-dose exam (<0.1 mGy)
Extremity radiograph	<0.001	Very low-dose exam (<0.1 mGy)
Abdominal radiograph	0.1–0.3	Low-to-moderate dose (0.1–10 mGy)
Lumbar spine radiograph	1.0–10	Low-to-moderate dose (0.1–10 mGy)
Intravenous pyelogram (IVP)	5–10	Low-to-moderate dose (0.1–10 mGy)
Computed tomography (CT) pulmonary angiography/chest CT	0.01–0.66	Low-to-moderate dose (0.1–10 mGy)
Limited pelvic CT	<1	Low-to-moderate dose (0.1–10 mGy)
Abdominal CT	1.3–35	High-dose exam (10–50 mGy)
Pelvic CT	10–50	High-dose exam (10–50 mGy)
Whole-body PET/CT with 18F-FDG	10–50	High-dose exam (10–50 mGy)

Data derived from [93].

## Data Availability

The original contributions presented in this study are included in the article. Further inquiries can be directed to the corresponding author.
